# Experiences of family and community care physicians and nurse residents in Madrid regarding their training during the COVID-19 pandemic: a photovoice study

**DOI:** 10.1186/s12909-026-08769-9

**Published:** 2026-02-17

**Authors:** Elena Polentinos-Castro, Raquel Sánchez-Ruano, Paloma Conde-Espejo, Miguel López-Miguel, Lucía Nuevo-Coello, María Minué-Estirado, Elena Valera-Bermejo, Isabel del Cura-González

**Affiliations:** 1 Research Unit, Primary Care Management, Madrid Health Service, Madrid, Spain; 2 Department of Medical Specialities and Public Health, Rey Juan Carlos University, Alcorcón, Madrid, Spain; 3 Network for Research on Chronicity, Primary Care, and Health Promotion (RICAPPS), ISCIII, Madrid, Spain; 4 Instituto de Investigación Sanitaria Gregorio Marañón, Madrid, Spain; 5 Foundation for Biosanitary Research and Innovation in Primary Care (FIIBAP), Madrid, Spain; 6 Las Ciudades Health Centre Multiprofessional Teaching Unit of Family and Community Care South, Madrid Health Service, Primary Care Management, Madrid, Spain; 7 Mª Jesús Hereza Health Centre, Multiprofessional Teaching Unit of Family and Community Care South, Primary Care Management, Madrid Health Service, Madrid, Spain; 8 José María Llanos Health Centre, Multiprofessional Teaching Unit of Family and Community Care Southeast, Primary Care Management, Madrid Health Service, Madrid, Spain; 9 Puerta Bonita Health Centre, Multiprofessional Teaching Unit of Family and Community Care Centre, Primary Care Management, Madrid Health Service, Madrid, Spain; 10 Ageing Research Center, Karolinska Institutet and Stockholm University, Stockholm, Sweden

**Keywords:** Community-Based Participatory Research, Photovoice, COVID-19, Professional Education, Internship and residency, Primary Health Care

## Abstract

**Background:**

The COVID-19 pandemic brought about sudden changes in healthcare demands, requiring additional healthcare professionals and altering the educational landscape for medical and nursing residents. This study aimed to explore the experiences and impact of the pandemic on the training of family and community medicine as well as family and community nursing residents.

**Method:**

A participatory qualitative study was conducted using the Photovoice methodology. Seven residents in family and community medicine and nursing in the Madrid region were purposively sampled. Participants took photographs and engaged in weekly sessions over five weeks to discuss and analyse their photos, which were grouped into themes and further categorized. Recommendations for institutions and program leaders were developed using an adapted logical framework approach.

**Results:**

The average age was 27 years, with 86% being female. Participants captured 96 photographs categorized into five themes (physical and emotional impact; impact on primary care system; hospital care ; social and environmental impact ; and impact on training). Through critical discussions on impact on training-related photos, seven emergent themes were identified: 1) COVID-19 monograph, buried knowledge; 2) Changing roles ; 3) Transition from face-to-face to online delivery format ; 4) Supervision; 5) Tutor as educator and caregiver ; 6) Advocacy ; 7) Family and nursing residents as agents of change. Subsequently, participants formulated seven recommendations that should be considered in similar situations.

**Conclusion:**

The COVID-19 pandemic has posed significant challenges in implementing medical education programs. It has had both formative and professional impacts on medical and nursing residents. These findings can aid in designing coping and educational strategies to navigate exceptional situations such as the one experienced during the pandemic.

**Supplementary Information:**

The online version contains supplementary material available at 10.1186/s12909-026-08769-9.

## Introduction

The COVID-19 pandemic forced the reorganization of healthcare systems worldwide, generally following the guidelines of the World Health Organization (WHO) [1]. In Spain, the third country with the most cases in Europe [2, 3], the outbreak of the pandemic in March 2020 saturated the capacity of the healthcare system and necessitated organizational changes at all levels of care [4]. According to the ENE-COVID study in May 2020, the seroprevalence for the entire country was 5.0%, reaching 11% in the Madrid region [5, 6]. In primary care in the Madrid region as of May 11, 2020, a total of 231,765 positive COVID-19 cases had been attended to, of which 88,707 (44%) required hospitalization and 7,306 (5.3%) were admitted to intensive care units [7].

This reorganization of healthcare systems also affected undergraduate health science students and professionals undergoing specialized healthcare training [8, 9]. Many of them had to take on unanticipated clinical activities not included in their training programs to cover the sudden need for additional workforce. In a study that surveyed orthopaedic residents from 29 countries, over 50% of them reported performing tasks outside their postgraduate training programmes [10]. In other cases, their programs were modified as in-person clinical rotations ceased in many areas [11], mainly due to the focus on COVID-19 and the limitation of non-COVID care [7]. After the first wave, a significant portion of training shifted from in-person to virtual to ensure COVID prevention measures and social distancing. Seminars and other didactic sessions that had been cancelled were then held virtually through online media [12]. Even residents in specialties such as radiology in the USA saw reduced training encounters, with most training being conducted online [13]. In Italy, neurology residents had fewer research hours during the first wave [14]. In most specialties, specific training took many months to recover [13, 15], which had an impact on their specialization training [16], with some cases expressing a desire to extend in training time [13]. However, positive effects have also been identified, considering that the pandemic gave them the opportunity to engage more in research, teamwork, and to better coordinate with colleagues [17, 18].

On the other hand, the incorporation of telephone consultations and new technologies for patient care required the acquisition of new skills and competencies not included in current training programs [19, 20].

In addition to the training impact, the experiences lived through the pandemic have led to significant psychological distress, with an increase in anxious-depressive symptoms [21], to occurred in healthcare professionals in general [22–27]. A decrease in sleep hours and quality, and worsening lifestyle habits such as increased consumption of simple sugars, has also been identified [28]. Regarding job satisfaction, quality of life, and perspective on the profession, different studies provide contradictory results [29, 30].

Different qualitative techniques allow us to approach the analysis of discourse and the experiences and perceptions of these professionals, including the Photovoice as a participatory action research approach. Photovoice has been previously used to explore educational experiences in health sciences [31] and to study the work impact of the COVID-19 pandemic on healthcare professionals in Canada [32] and Spain [33].

Spain’s National Health System (SNS) is publicly funded, providing universal healthcare coverage free of charge at the point of use. In Spain, the specialty training system for health professions follows a competency-based training model. [34]. For specialists in Family Medicine (primary care training or GP in UK) and Family Nursing, the postgraduate training program duration is 4 and 2 years respectively, conducted in accredited primary health care centres and hospitals [35, 36]. This postgraduate training and professional performance in both healthcare settings have given family medicine and nursing in training a distinctive character regarding the impact and experiences during the pandemic.

The aim of this study is to understand the experiences, perceptions, and impact of the pandemic on the postgraduate training of Family and Community Medicine and Nursing.

## Methods

### Study design

We conducted a qualitative study using photovoice, a participatory action research technique. The study took place during the first semester of 2021 in primary care settings in the Madrid region, with the aim of exploring the experiences and impact of the pandemic on the mental health of primary care professionals using mixed methods [37]. For the preparation of this article, we followed the recommendations of the Standards for Reporting Qualitative Research [38], Supplement 1.

We conducted this study in accordance with the Declaration of Helsinki and ethical approval was obtained from the Ethics Committee of Hospital de la Princesa, Madrid (MIND/COVID-19-19/4403, 2021).

### Participants and setting

To recruit participants for the photovoice sessions among medical and nursing residents, we considered their year of training and professional profile (medicine/nursing) using an intentional sampling strategy [39]. We formed a group of 7 male and female medical and nursing residents, MIR (Medical Interns) and EIR (Nursing Interns) from the Family and Community Care Teaching Units in Madrid, who had been in their training program for at least 6 months and were available to attend the 5 photovoice sessions (Table 1). Participants were fully informed about the project and signed a consent form. There was no financial incentive to participate in the study; however, participants received a portrait taken by a professional photographer as a token of appreciation for their participation.

**Table 1:** Characteristics of the participants

	**Sex**	**Age**	**Health field**	Year of residency*
Participant 1	Female	26	Family medical resident	First year
Participant 2	Male	26	Family medical resident	First year
Participant 3	Female	25	Nurse medical resident	Second year
Participant 4	Female	27	Family medical resident	Second year
Participant 5	Female	27	Family medical resident	Third year
Participant 6	Female	27	Nurse medical resident	Second year
Participant 7	Female	28	Family medical resident	Fourth year

^*^Family nurse residency: 2 years long, Family medicine residency: 4 years long

### Data collection

#### Phase 1: photovoice process

The group met weekly for 5 weeks, with sessions lasting approximately 2 hours each. Each session was facilitated by a sociologist expert in photovoice method, and two-family physician observers with training in medical anthropology and epidemiology, as well as a photographer.

During the first session, a brief introduction to the project and the content of each of the five sessions was provided. Participants filled out informed consent forms and a demographic questionnaire. Following this, basic photography training was conducted, covering topics such as ethics and responsibilities of being a photographer, along with instructions on the photographic tasks for the project. Participants took photos with their phones. At the end of the first session, participants were asked to photograph their experiences, feelings, and the impact of the pandemic on their training. While participants could take as many photos as they deemed appropriate, they were only allowed to bring a maximum of five photos to the following sessions to focus the discussion better [40]. A modified version of the mnemonic method SHOWED [41] was used to guide discussions about the photographs, with the following questions: 1. What does the photograph show? 2. What is the story behind? 3. How does this relate to your work/training as a resident?

In the second session, each participant brought a maximum of five photos. Facilitators only intervened to clarify doubts or to engage participants who were not actively participating in the discussions. In sessions 2, 3, and 4, medical and nursing residents could contribute new photos or continue discussions from previous sessions. In session 5, participants categorized their photos into themes and decided which photos would be selected in the final version.

#### Phase 2: development of recommendations

Once ideas were coded into themes and categories, participants created a problem analysis tree and identified recommendations for healthcare institutions using an adapted logical framework approach [42]. This approach is commonly used to identify community needs, describe problems and desired improvements, and propose possible solutions [43].

The problem tree consisted of a trunk representing the main problem (e.g., monographic COVID-19 care). From this trunk, several tree knots represented specific problems (difficulty in acquiring specialty-specific competencies); from the tree knots, hanging roots represented causes (focus on acute care over chronic care). Once the tree was constructed, participants reformulated it into a solutions tree [42]. For example, specific problems turned into solutions (provide supplementary training), and roots became policy recommendations (define an action plan for recovering competencies and acquiring skills).

### Data analysis

Data included photographs with their photographic narratives, reflections made by participants during discussions, recommendations they drafted, and notes taken by observers during group sessions. Participants themselves identified common codes and themes arising from photographs, and during the final group session, medical and nursing residents were asked to reflect and assign photos to categories, choosing a representative photo for each category. Collaboratively with the researchers, categories were grouped into broader themes, and data were analyzed using a deductive content analysis approach, a method "for qualitative data analysis in which the researcher goes back and forth repeatedly between empirical data and abstract concepts or theories," and these themes were agreed upon with the participants.

Researchers classified recommendations into two types related to formative or occupational aspects. Researchers made decisions at each phase of the study and in data analysis (e.g., identifying key themes and descriptive analysis) and attempted to reach consensus in result reporting with the entire team. Family physician researchers reflected on potential implications as they shared spaces and experiences with participants during the pandemic.

## Results

A total of 96 photos were collected and grouped into five categories: physical and emotional impact; impact on the primary care system; hospital care; social and environmental impact; and impact on training. In this article, we focus specifically on the categories and findings related to training, as postgraduate specialist healthcare training is the primary objective of the study. While the remaining categories form part of a broader analysis conducted among healthcare professionals in general, the present manuscript reports only those results that directly address the impact of the pandemic on residents in training.

Within the category related to the impact on training, seven main themes were identified: 1) COVID-19 monograph, buried knowledge; 2) changing roles; 3) from face-to-face to an online delivery format; 4) supervision; 5) tutor as educator and caregiver; 6) demands; and 7) family medicine and nursing residents as agents of change. Representative photos were selected for each of these themes, which are detailed below.

### Theme 1: COVID-19 monograph, buried knowledge

**Fig. 1:**
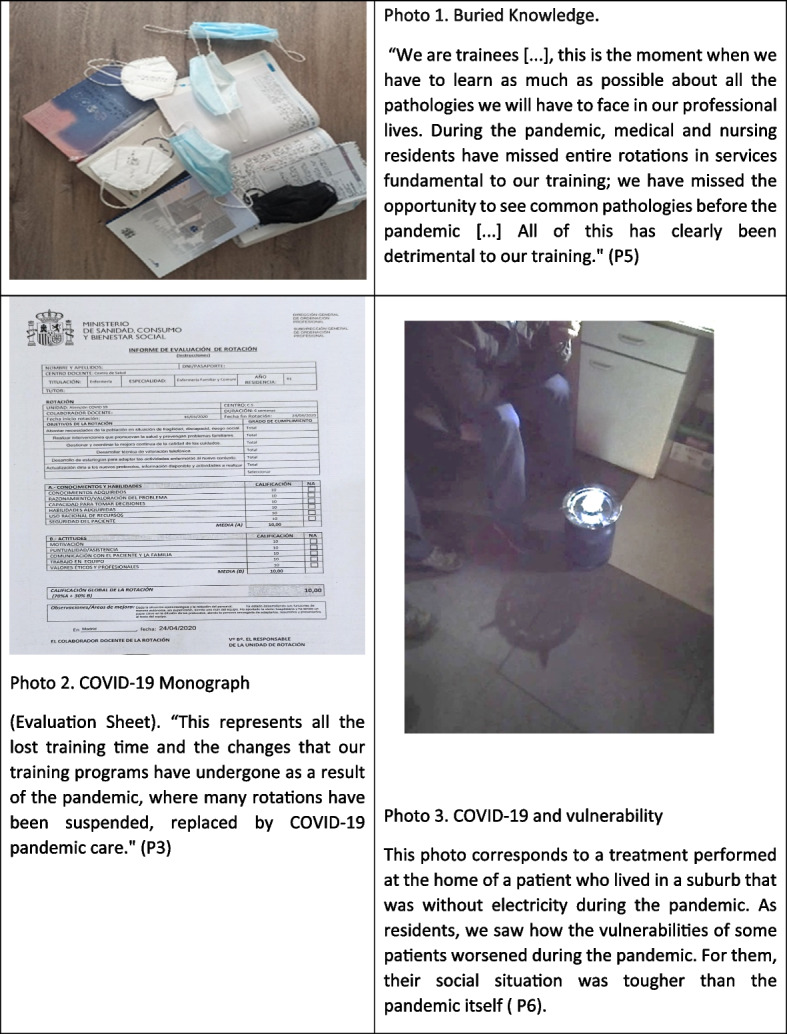
COVID-19 monograph, buried knowledge

At the beginning of the pandemic, most medical and nursing residents were relocated from their planned rotations to primary care centres and/or hospital emergency departments to contribute to the pandemic response. They considered this relocation affected their training. The care for chronic patients and the active follow-up of these patients, which are fundamental aspects of Primary Care, practically disappeared in the early waves of the pandemic in most healthcare facilities (Figure 1).

*"People only came for acute problems they considered serious and non-deferrable. Chronic patients endured and endured until they got worse. We didn't see the typical consultations for chronic patient management in primary care" (P2). "Due to the pandemic situation, very few people come in person to consultations out of fear, and therefore, we have lost a lot of training as medical and nursing residents" (P5).*

### Theme 2: changing roles

**Fig. 2:**
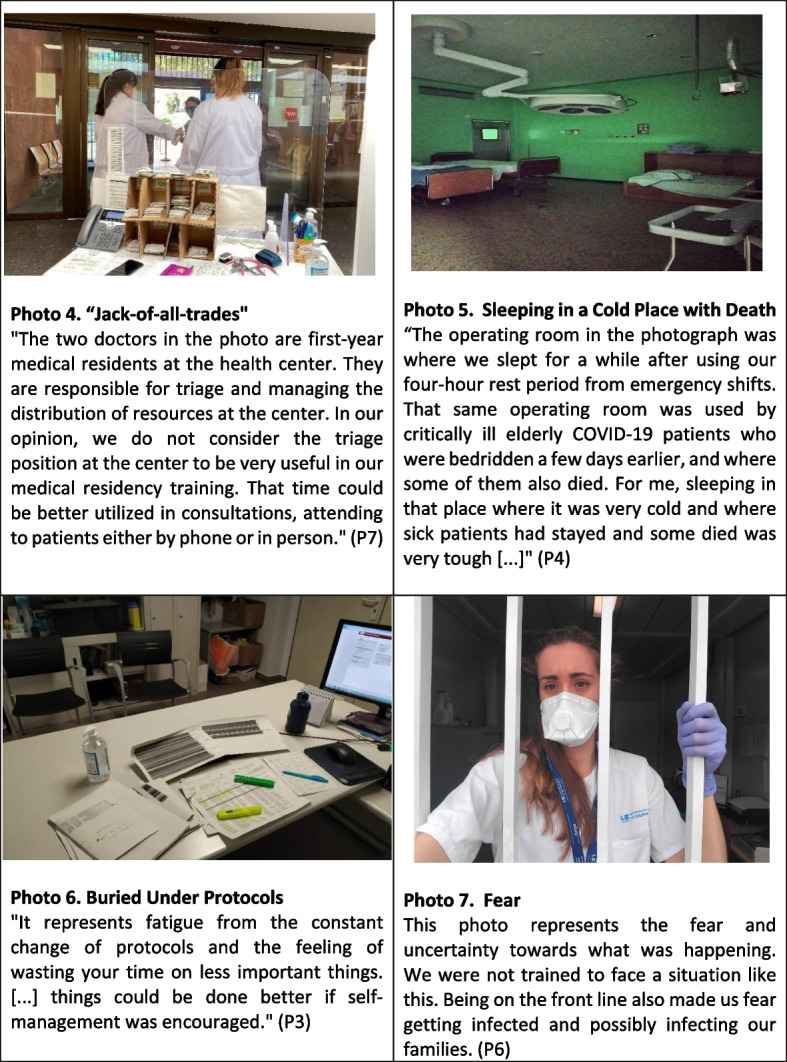
Changing roles

Medical and nursing residents, especially those in the first half of their training, had to adapt to continuous changes in their work environment and to new tasks, to meet the organization's needs. They worked in hospital emergency departments, rural areas, their reference primary care health centre, and Madrid's pandemic field hospital. They were frequently relocated based on staffing needs, which led to frequent changes in their team members. Additionally, new COVID-19 protocols were periodically published for each of these work areas, which medical and nursing residents had to learn, affecting the time they could dedicate to studying other pathologies (Figure 2).

“*The family medicine and nursing residents were working in the health centre, hospital, and also doing rural shifts. Protocols were constantly changing at each location, and we were expected to know them all, but then we didn't have time to study anything else." (P4)*

### Theme 3: from face-to-face to an online delivery format

**Fig. 3:**
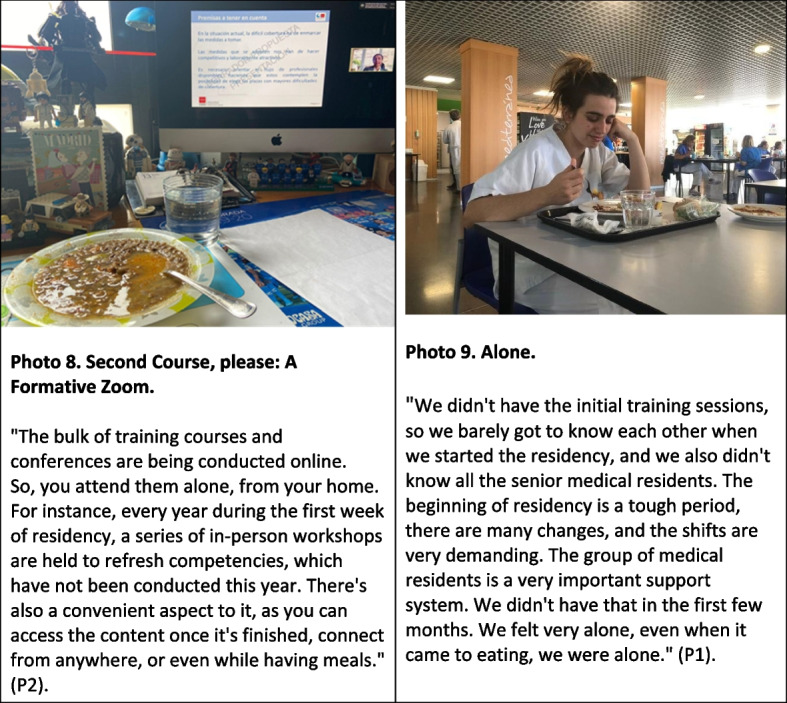
From face-to-face to an online delivery format

Due to the risk of contagion in large gatherings, especially considering the high exposure to COVID-19 among healthcare personnel, the online format replaced the in-person format for all training activities. While medical and nursing residents acknowledged that the online format offered some advantages, it meant losing an important arena for sharing experiences and support. For first-year medical and nursing residents, they also lost the spaces that traditionally provided the opportunity to meet peers from the same year and previous years, which allowed them to build a social support network with their colleagues (Figure 3).

### Theme 4: supervision

**Fig. 4:**
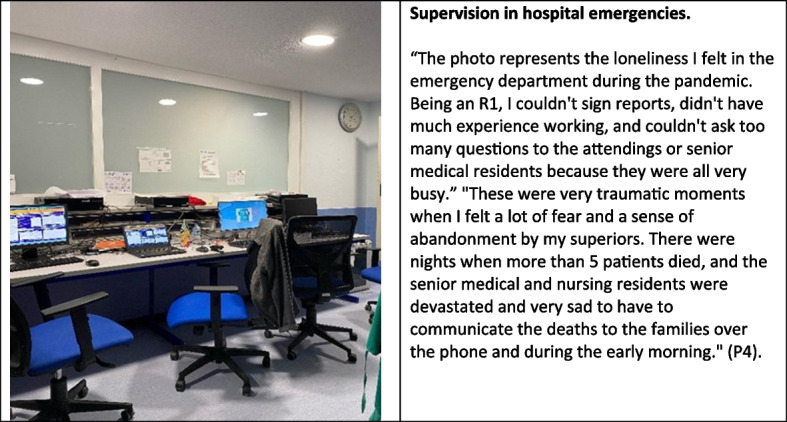
Supervision

In the case of hospital shifts, medical residents reported less supervision in hospital emergency departments during the pandemic, especially in hospitals that were overwhelmed, which was not identified in other healthcare settings such as primary care centres. Nurses, on the other hand, considered that their supervision was adequate in both healthcare settings. These situations occurred in a context where the healthcare pressure was overwhelmed, leading to less supervision for medical residents in emergency departments, which generated feelings of insecurity and discomfort among residents (Figure 4).

### Theme 5: tutors as educators and caregivers

**Fig. 5:**
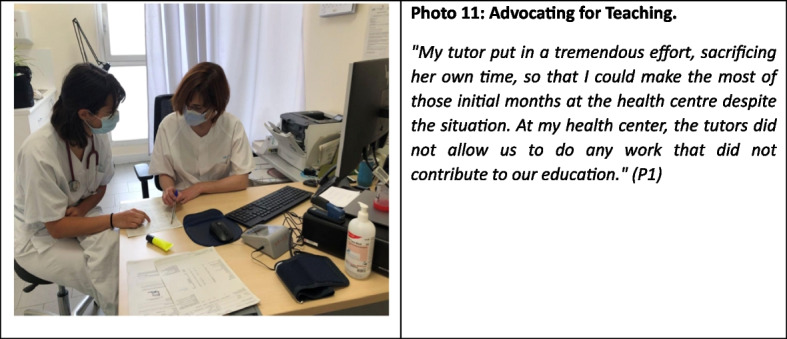
Tutors as educators and caregivers

In the face of feelings of vulnerability and loss of training opportunities overall, the medical and nursing residents identified Primary Care tutors as a caring figure and guarantor of their education (Figure 5).

"*The protection of my tutor, who is always looking out for me.*" (P3)

*“The tutors played a central role in protecting our education during the pandemic. The workload, telephone consultations, staff absences, rescheduling of appointments, or the high number of COVID-19-related consultations have been significant obstacles to our learning. In response to this situation, the tutors at the health centres made a tremendous effort to ensure educational spaces whenever possible. For me, the constant support from my tutor has helped me maintain motivation and enthusiasm in a very challenging time for primary care." (P4)*

Throughout the photovoice process, participants expressed their frustration regarding the training they received and the psychological exhaustion of being on the front lines without adequate preparation. However, they also acknowledged feeling useful and supported by their tutors and colleagues in this unprecedented crisis for them.

### Theme 6: claims

**Fig. 6:**
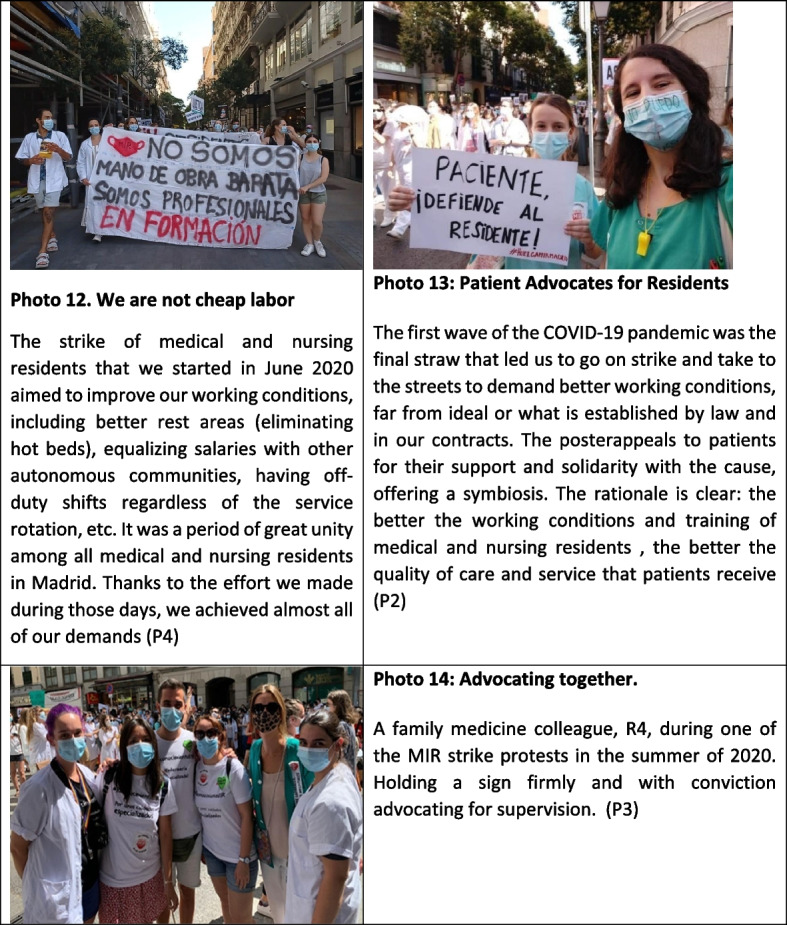
Claims

The pandemic, especially during the first wave, accentuated the labor and training issues that medical and nursing residents were already facing (Figure 6). In July 2020, medical and nursing residents in the Community of Madrid launched an open-ended strike to protest their working conditions. They called for fair wages, adequate rest after on-call shifts, proper supervision, and decent conditions during those shifts — including suitable rest areas. After five weeks, they reached a preliminary agreement with the Regional Health Ministry. While it didn’t fully meet all their demands, it marked a meaningful step forward. On a broader level, the strike also sought to raise public awareness about the vital role residents play during their years of training, especially in providing coverage for emergency on-call duties within the healthcare system.

"*We are being used as cheap labor; there are services and hospitals that function entirely thanks to medical and nursing residents. However, we are trainees, and learning should be the priority*"

### Theme 7: family medicine residents as agents of change

**Fig. 7:**
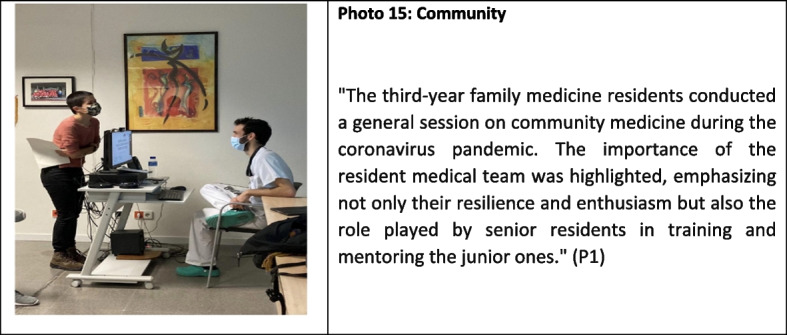
Family medicine residents as agents of change

The medicine and nursing residents who participated in the study identified their fellow residents as a source of resilience and motivation, maintaining a commitment to the family and community approach, as well as emphasizing the importance of defending the educational and labor rights (Figure 7).

The photovoice technique as a research-action method generated a reflective process that led to critical awareness and constituted a learning experience that involved making proposals for change. Following the logical framework approach, participants identified seven policy recommendations related to specialty healthcare training. Table 2 provides an overview of the entire set of recommendations made by the participants.

**Table 2:** Residents’ recommendations to policy makers

Theme	Question of improvement	Recommended strategy
COVID-19 monography	How to improve the acquisition of competencies other than COVID-19 patient management?	Establish a programme with specific competencies to be adquired per year of residency and reinforce the figure of a head of studies to guarantee its fulfilment.
Changing roles	How to improve the role of the resident in daily labour?	Residents should be represented and actively participate in workers´ committee/meetings where tasks and functions are decided
From face-to-face to an online delivery format	How to improve online training?	Online training offers a series of strengths and opportunities, but spaces and security conditions must be provided for face-to-face training when necessary or when requested. It must be possible to make up for lost training by adding new editions.
Supervision	How to improve resident supervision?	There is a need to hire more staff to improve the ratio of faculty members to residents to allow for adequate supervision.
Tutor as trainer and resident caregiver	How to promote the figure of the tutor as trainer and caregiver?	Reduce the workload of tutors so that they can devote more time and resources to the training of residents and work closely with them.
Claims	How to monitor compliance with working conditions?	Establish a committee to investigate and monitor compliance according to the agreements reached.
Family residents as drivers of change	How to promote the figure of the family resident as a driver of change?	To promote the implementation of training, research and community health activities during working hours.

## Discussion

In the study, seven categories of analysis were identified, and seven recommendations were prioritized in a participatory action research process.

The training program for family medicine and nursing residents is largely based on progressively acquiring competencies and responsibilities, practicing medicine or nursing with full supervision at the initial stages and a gradual reduction in supervision as training [35, 36].

During the early stages of the pandemic, this supervision was quite affected due to the exceptional nature of the situation. Like other healthcare professionals, they had not experienced such a complex professional situation before, but in their case, it was compounded by their limited previous professional experience before the pandemic and within the context of a training process. This was more pronounced for residents in their first two years of specialty training. The supervision and mentoring of residents has been a topic that reflects a great variability among different environments and professional profiles, with nursing residents generally feeling more supported. Regarding medical residents, there is a higher level of supervision by their tutors in the primary care setting compared to hospital tutors, although this has also depended on the respective hospital. This contrasts with findings from another study in the United States, where most residents reported feeling adequately supervised [44]. The lack of supervision, although a common complaint among medical residents, was likely exacerbated not only by the overload of the entire healthcare system but also by a "disconnect" between educators and medical and nursing residents due to high staff turnover in all services.

One of the most unfavourable conditions identified included insecurity in the professional role when working on the front line and the fear of the situation and managing uncertainty. Unlike previous epidemics such as Ebola, where trainee professionals were removed from the front line [45], this was not the case in this instance, medical and nursing residents were frontline workers. As they were generally young and healthy, they were at a lower risk of developing a severe illness in case of infection. The need for personnel redistribution in areas with high demand and staff shortages due to the increasing number of patients and infections among healthcare workers [46] led to many medical and nursing residents being reassigned to other services during critical moments of the pandemic [12, 47]. This reassignment to different services affected family and community medicine residents more than other specialties because of their more transversal and multidisciplinary training, allowing them to perform professionally in primary care centres, hospitals, and out-of-hospital emergency services. All of this was associated with an increase in work hours, which resulted in less time dedicated to specialty training, similar to what has been described in other studies [12, 14, 48], but other studies conducted in the United States reported a decrease in face-to-face working hours in some specialties [49], which in some cases was partially compensated by an increase in the number of hours worked from home [50].

During the early waves of the pandemic, learning environments were severely affected and deteriorated, consistent with findings from studies conducted in Spain and other countries in our region [12, 47, 51, 52]. In the USA and the European Union, significantly less time was spent in hospitals, clinics, and operating rooms. In our study, it was the opposite, residents remained on the front lines both in hospitals and primary care for more hours, unlike other residents who spent more time using telemedicine, worked from home during the pandemic, and were able to dedicate time to research projects, educational conferences, non-medical hobbies, and reading The proximity to the population and providing home care also made them, along with their supervisors, witnesses to situations of social vulnerability that were accentuated during the pandemic [53].

Virtual care has been another key point in the process. Virtual care is a work method that residents in medicine and nursing, as well as experienced professionals, were not accustomed to in general, and there were no competencies regarding it in the training programs of residents in medicine and nursing [54, 55]. Up until the pandemic, the training of residents in medicine and nursing in clinical encounters and communication skills had been focused on acquiring abilities for in-person care in various healthcare settings.

Additionally, this change led, similar to what has been described in other studies, to a limitation or disappearance of physical examinations, substantially reducing the capacity for learning and teaching. Similarly, the time constraints imposed by virtual modalities translated into fewer teaching moments for residents [56]. This was worsened by the fact that the patient profile primarily consisted of respiratory cases infected with COVID-19, as described in the "COVID-19 Monograph" category, which limited their learning opportunities. While some studies have investigated the perspectives of patients and providers during the transition to virtual care, few have explored the impact of virtual care on the education of medical and nursing residents [57, 58].

Besides changing the way outpatient healthcare was delivered, virtual care also altered how tutors and educators teach, and modified the learning experiences of medical and nursing residents [59]. Regarding virtual education, the findings in our study align with other research; overall, online education has been viewed as a positive tool by medical and nursing students and residents [60–62]. Regarding the negative aspects of online education, our results identified a lack of interaction in the learning process with the group of peers and the perception that virtual activities did not match the educational experience of hands-on learning in some areas. A similar sentiment is captured in the study by Kunaviktikul et al: 'But nobody answered, all silent... This is very different from when we were physically [in class], as everyone was very active in participating... I could see their expressions... I feel lonely...' [31]. Distance learning requires technological skills from both students and faculty, as well as adaptation to individual learning needs [62], aspects that were not fully implemented in many cases at the start of the pandemic."

During times of organizational chaos and continuous change, complaints tend to be directed at the top of the hierarchy for their inability to adapt quickly [63]. Meanwhile, colleagues in closer proximity emerge as support and reference figures, with micro-organization among them being a structure with greater capacity to adapt to the new real needs of daily practice.

The pre-pandemic discontent with their working conditions was exacerbated by the pandemic, leading to a significant surge in advocacy and demands. For family medicine and nursing residents, their dissatisfaction cannot be separated from the weakened state of primary care in Spain, which has structural problems that were exacerbated and highlighted during the pandemic [51].

Focusing on the proposed recommendations made by the participants, two groups can be identified. The first group focuses primarily on the pandemic, highlighting the need for participation in exceptional situations such as the one experienced, improved work planning and decision-making through representatives, the definition of competencies according to the year of training, and compensation for lost training. Recommendations from the second group go beyond the context of the pandemic.

These include improvements in spaces and safety conditions for face-to-face training complemented with virtual training; ensuring direct supervision especially in the early years; reducing the workload of tutors so they can devote more time and resources to resident training; monitoring compliance with the agreed labor conditions after the strike; and promoting the implementation of training, research, and community health activities during working hours."

### Strengths and limitations

The Photovoice technique has allowed residents of family and community medicine and nursing to be protagonists in a participatory process. The Photovoice methodology relies on the researcher's analysis and interpretation; however, in addition to being widely used to characterize the urban environment and citizen perception, it has also been an excellent tool for exploring the perception of healthcare professionals about providing care to different population groups [41, 64, 65]. This technique allowed them to metaphorically represent what had the most significance for them. It guided researchers on which aspects are more relevant from the participants' perspective. Their debates and analysis around the impact of the pandemic on their training generated a set of recommendations to improve training aspects and potential deficits in the future. We consider that the use of photographs has been an advantage to evoke deeper elements of the lived experience and to help unlock memories that would have been more difficult to verbalize. This strength of the technique has been analyzed in comparison with word analysis or narratives [66].

In our study, the use of a participatory diagnostic approach enabled us to inform decision-makers in training and healthcare planning of the recommendations developed. In addition to generating new knowledge, the participating medical and nursing residents have experienced a more enriching process of reflection and learning by sharing their experiences with others. Simultaneously, it has provided an opportunity to share experiences and stories, which has had a therapeutic effect.

The generalization of the results from our study is limited to environments that share a similar training program and healthcare system model as in Spain. The majority participation of women in the study could be considered a bias, although it is quite consistent with widely feminized professions.

During the COVID-19 pandemic, postgraduate specialist training experienced significant losses in supervised, experiential learning, while rapidly adapting through online education and virtual care. A key learning is the vulnerability of training programmes during health system crises and the central role of supervision, mentoring, and tutors in sustaining training quality. Future crisis preparedness should strengthen trainee participation, planning, and compensation mechanisms, while structurally reinforcing supervision and training conditions beyond emergency contexts.

## Conclusions

The COVID-19 pandemic has posed significant challenges in the implementation of medical education programs. For medical and nursing residents, it has had an impact both in terms of training and work. These results and the recommendations developed can help design coping and training strategies to deal with exceptional situations like the one experienced.

## Supplementary Information

Supplementary Material 1.

## Data Availability

The datasets supporting the conclusions of this article are included within the article and its additional files.
